# *Porphyromonas gingivalis* within Placental Villous Mesenchyme and Umbilical Cord Stroma Is Associated with Adverse Pregnancy Outcome

**DOI:** 10.1371/journal.pone.0146157

**Published:** 2016-01-05

**Authors:** Sizzle F. Vanterpool, Jasper V. Been, Michiel L. Houben, Peter G. J. Nikkels, Ronald R. De Krijger, Luc J. I. Zimmermann, Boris W. Kramer, Ann Progulske-Fox, Leticia Reyes

**Affiliations:** 1 Department of Pediatrics, Maastricht University Medical Center, Maastricht, the Netherlands; 2 School for Mental Health and Neurosciences (MHeNS), Maastricht University, Maastricht, the Netherlands; 3 School for Public Health and Primary Care (CAPHRI), Maastricht University, Maastricht, the Netherlands; 4 Division of Neonatology, Erasmus University Medical Center–Sophia Children’s Hospital, Rotterdam, the Netherlands; 5 Department of Pediatrics, Wilhelmina Children’s Hospital, University Medical Center Utrecht, Utrecht, the Netherlands; 6 Department of Pathology, University Medical Center Utrecht, Utrecht, the Netherlands; 7 Department of Pathology, Erasmus University Medical Center, Rotterdam, the Netherlands; 8 School for Oncology and Developmental Biology (GROW), Maastricht University, Maastricht, the Netherlands; 9 Department of Oral Biology, Center for Molecular Microbiology, University of Florida, Gainesville, Florida, United States of America; 10 Department of Pathobiological Sciences, University of Wisconsin-Madison, Madison, Wisconsin, United States of America; Tokyo Medical and Dental University, JAPAN

## Abstract

Intrauterine presence of *Porphyromonas gingivalis* (Pg), a common oral pathobiont, is implicated in preterm birth. Our aim was to determine if the location of Pg within placental and/or umbilical cord sections was associated with a specific delivery diagnosis at preterm delivery (histologic chorioamnionitis, chorioamnionitis with funisitis, preeclampsia, and preeclampsia with HELLP-syndrome, small for gestational age). The prevalence and location of Pg within archived placental and umbilical cord specimens from preterm (25 to 32 weeks gestation) and term control cohorts were evaluated by immunofluorescent histology. Detection of Pg was performed blinded to pregnancy characteristics. Multivariate analyses were performed to evaluate independent effects of gestational age, being small for gestational age, specific preterm delivery diagnosis, antenatal steroids, and delivery mode, on the odds of having Pg in the preterm tissue. Within the preterm cohort, 49 of 97 (51%) placentas and 40 of 97 (41%) umbilical cord specimens were positive for Pg. Pg within the placenta was significantly associated with shorter gestation lengths (OR 0.63 (95%CI: 0.48–0.85; p = 0.002) per week) and delivery via caesarean section (OR 4.02 (95%CI: 1.15–14.04; p = 0.03), but not with histological chorioamnionitis or preeclampsia. However, the presence of Pg in the umbilical cord was significantly associated with preeclampsia: OR 6.73 (95%CI: 1.31–36.67; p = 0.02). In the term cohort, 2 of 35 (6%) placentas and no umbilical cord term specimens were positive for Pg. The location of Pg within the placenta was different between preterm and term groups in that Pg within the villous mesenchyme was only detected in the preterm cohort, whereas Pg associated with syncytiotrophoblasts was found in both preterm and term placentas. Taken together, our results suggest that the presence of Pg within the villous stroma or umbilical cord may be an important determinant in Pg-associated adverse pregnancy outcomes.

## Introduction

Every year 15 million babies are born preterm [1], one million of these babies die due to complications of preterm birth [2]. Those who survive are at risk for serious morbidity such as adverse neurodevelopmental outcomes [3], chronic lung disease (e.g. bronchopulmonary dysplasia) [4], and asthma [5]. Intrauterine infection is one of the most common causes of preterm deliveries [1, 3, 6], and ascending infection into the amniotic cavity is considered the core mechanism [3]. Recent studies indicate that oral bacteria, which comprise a highly diverse microbiome, may also play an important role in the pathogenesis of preterm delivery [3, 7–11].

There are two principle biological mechanisms whereby periodontal bacteria are thought to promote adverse pregnancy outcomes [11]. The first model refers to an indirect pathway through which pro-inflammatory mediators released from damaged periodontal tissues reach the fetal–placental unit via the circulation. The second model proposes that microorganisms and/or their components directly reach the fetal–placental unit via hematogenous dissemination from the oral cavity or, less likely, by ascending route from the lower genitourinary tract. Although these models form the basis of the current periodontal treatment guidelines for pregnant women, they do not fully address the mechanisms by which certain periodontal bacteria affect obstetric outcomes [11, 12]. For example, periodontal therapy during pregnancy has not made a significant impact on the reduction of preterm birth [11, 12]. Thus, additional studies are needed in order to better identify at risk populations, refine current therapeutic policies, and/or develop new modes of therapy.

*Porphyromonas gingivalis* (Pg) is a Gram-negative anaerobic asaccharolytic bacterium and common pathobiont of the oral cavity worldwide [13, 14]. Pg oral colonization rates range between 10 to 25% in healthy adults and 79 to 90% in adults with periodontal disease [14]. Pg is also implicated in a diverse array of pregnancy complications including low birth weight, intrauterine growth restriction, preeclampsia, and spontaneous preterm birth [9, 15–18]. Pg is considered a keystone species of periodontal disease because it produces an array of virulence factors that subvert host immunity and promotes a persistent inflammation [13]. Through this process Pg actually enables the emergence of dysbiotic oral communities that enhance disease severity [13]. Under these circumstances Pg may promote preterm delivery via the indirect pathway [11].

There is emerging evidence that Pg may contribute to adverse pregnancy outcomes by directly invading maternal-fetal tissues. In women with preeclampsia, Pg detection rates within the uterine compartment range between 30 and 92%; with prevalence being highest in studies that sampled the decidua/placental basal plate [15, 17, 18]. In rodents, monotypic infection of the utero-placental tissues produces fetal growth restriction, mild chorioamnionitis, endometrial arteritis, utero-placental thrombosis/hemorrhage with disruption of placental architecture, and increased production of pro-TH1 cytokines such as TNF-α, IFN-γ, IL-1, IL-12, and IL-17 in placental tissues [19–22]. As compelling as these findings are, the circumstances or mechanisms by which intrauterine infection with Pg promotes adverse pregnancy outcomes in women remain elusive. For instance, Pg has also been found in the placental tissue of women with normal pregnancies, albeit at a lower microbial load and lower frequency than women with preeclampsia or preterm birth [15–18].

Although maternal periodontitis is considered the source whereby oral bacteria gain entry into the circulation and reach the uterine compartment, clinical measures of periodontal disease cannot adequately reflect the infectious or inflammatory burden that is actually present in the pregnant women [11]. In this scenario, it is difficult to establish clear associations between the presence of oral bacteria at the maternal-fetal interface and preterm delivery. Therefore, the focus of this study was to determining if the presence of Pg within the placenta or umbilical cord was linked to adverse pregnancy outcome. We utilized an antibody based assay to determine if the *in situ* presence of Pg within placental and umbilical cord sections from very preterm infants (25 to 32 weeks gestation) [1] was different from normal term deliveries. In addition, a goal was to establish if Pg within placental and umbilical cord sections was linked to specific preterm pathology such as histologic chorioamnionitis (HC); histologic chorioamnionitis with funisitis as an indicator of fetal involvement (HCF); preeclampsia (PE); and preeclampsia with hemolysis, elevated liver enzymes, and a low platelet count (HELLP-syndrome). Based on the results of this study, we propose that the location of Pg within the villous stroma or umbilical cord may be an important factor in Pg-associated adverse pregnancy outcomes.

## Materials and Methods

### Ethical aspects

Specimens were prospectively collected with written parental consent and appropriate Institutional approval. The Rotterdam study was approved by the Medical Ethics Committee for Research on Human Subjects of the Erasmus University MC [23]. The Utrecht study was approved by the ethical review board of the Wilhelmina Children’s Hospital/University Medical Center Utrecht [24]. *In situ* detection of Pg was performed on de-identified sections with approval of the University of Florida Institutional Review Board.

### Patient and sample inclusion

**Fig 1:**
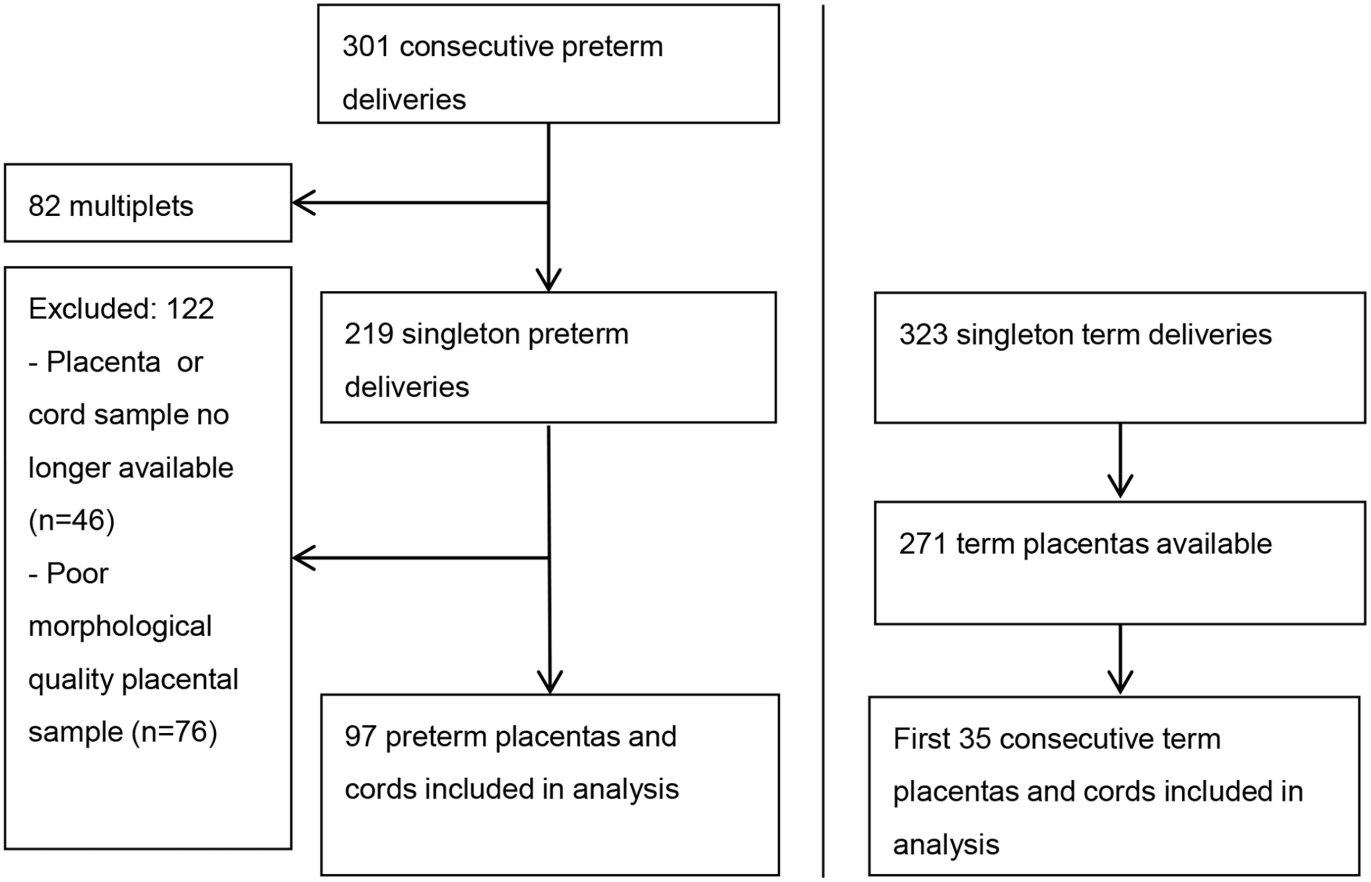
Flow chart of sample inclusion.

Fig 1 illustrates the inclusion/exclusion scheme for both preterm and term samples. The preterm cohort (≤ 32 weeks gestation) consisted of newborns born in the Erasmus Medical Center-Sophia Children’s Hospital, Rotterdam, The Netherlands, between May 2001 and February 2003 [23]. Patient characteristics of included (Table 1) and excluded preterm subjects are summarized in S1 Table. The term cohort consisted of newborns born following spontaneous onset of labor and vaginal delivery in the outpatient labor and delivery ward of the Wilhelmina Children’s Hospital in Utrecht, The Netherlands, between January 2006 and November 2007 (Table 1) [24]. In both cohorts, infants were excluded if major congenital abnormalities were present.

**Table 1:** Clinical characteristics of included subjects.

	Preterm newborns (n = 97)	Term newborns (n = 35)	*P* value
Maternal age (years)	31 ± 6	31 ± 5	0.79
Gravidity	2 (1–3)	2 (1–3)	0.99
Parity	0 (0–1)	1 (0–2)	0.60
Gestational (weeks)	29 ± 2	40 ± 1	<0.001
Full course antenatal steroids	70 (72%)	0	<0.001
PPROM	26 (27%)	0	0.001
Caesarean section	65 (67%)	0	<0.001
Placenta weight (grams)	260 ± 85	473 ± 78	<0.001
Preterm reference group	17 (18%)	28 (80%)	<0.001
HC	18 (19%)	6 (17%)	
HCF	23 (24%)	1 (3%)	
Preeclampsia	14 (14%)	0	
Preeclampsia + HELLP	25 (26%)	0	
Male gender	56 (58%)	20 (57%)	0.95
Birth weight (grams)	1162 ± 340	3501 ± 436	<0.001
SGA	23 (24%)	0	0.002
In-hospital mortality	13 (13%)	0	0.025

Numbers represent number and percentage of newborns or mothers in which characteristic is present for categorical data; median and interquartile range for ordinal data; and mean ± SD for continuous data. Differences between the included preterm and term subjects were tested using Chi-square, Mann-Whitney U and Student t-test, as appropriate. Abbreviations: PPROM, preterm premature rupture of membranes; HELLP, hemolysis, elevated liver enzymes and low platelet count; HC, histologic chorioamnionitis; HCF, histologic chorioamnionitis with funisitis; SGA, small for gestational age.

Gestational age was extracted from the medical records, and the determination of the method of ascertainment was thus at the discretion of the attending obstetrician. For each delivery we recorded the following: maternal age, gravidity, parity, antenatal steroid administration (betamethasone 12 mg intramuscularly, repeated after 24 hours), preterm premature rupture of membranes (PPROM), placental weight and delivery mode. We also documented whether there was a clinical diagnosis of preeclampsia and whether or not this diagnosis was accompanied by HELLP-syndrome. Preeclampsia was defined by new onset hypertension after 20 weeks gestation, accompanied by proteinuria (>300mg/24 hours). Hypertension was defined as a resting blood pressure >140/90 mmHg or mean arterial pressure >105 mmHg recorded on at least two separate occasions [23].

Collected neonatal data included 1, 5 and 10 minute Apgar-scores, infant gender, perinatal mortality and birth weight. A birth weight less than the 10^th^ centile for gestational age was considered small for gestational age (SGA) [23].

### Tissue collection and placental histology

Placentas, and umbilical cords were collected immediately after each delivery. Placentas with membranes and umbilical cord were fixed for 24–48 hours. The placenta was cut in slices of 1–2 cm to determine the presence of macroscopical lesions. Two umbilical cord blocks (one from the fetal and one from the placental end of the umbilical cord), and three blocks of normal placental parenchyma (sampled from the central area of the placenta) were embedded in paraffin, processed and stained with hematoxylin and eosin.

Placental and umbilical cord samples were assessed for signs of HC by determining the presence of polymorphonuclear cells present in the chorionic plate or membranous chorionic connective tissue and/or the amnion, as suggested by the Amniotic Fluid Infection Nosology Committee [25]. The diagnosis of HCF included any of the following features: chorionic vasculitis, umbilical phlebitis, umbilical (pan) vasculitis, (sub-acute) necrotizing funisitis, or concentric umbilical perivasculitis [25]. Ninety-seven preterm placental and cord samples from singleton deliveries were of acceptable morphological quality for immunofluorescent analysis.

### Immunofluorescent studies

Two serial sections from each placenta and umbilical cord specimen were examined for the presence of Pg antigen. Pg antigen was detected with a rabbit polyclonal antiserum diluted at 1:2000 [26], which was confirmed to recognize several strains of Pg [26], and validated for specificity (S1A Fig). With each experiment, a separate section that was incubated with pre-immune serum from the same rabbit host was included to define non-specific binding (S1B Fig).

Trophoblasts were detected with a cytokeratin 7-specific mouse monoclonal antibody (Abcam^®^, Cambridge, MA) [27]. Myofibroblasts were detected with a vimentin-specific mouse monoclonal antibody (Abcam^®^, Cambridge, MA) [28]. Goat anti-rabbit ALEXA 594 (absorption 590, emission 617) and goat anti-mouse ALEXA 488 (absorption 495, emission 519) (Life Technologies^™^, Grand Island, NY) were used as secondary antibodies. Cell nuclei were stained with 4′,6-diamidino-2-phenylindole (DAPI). All stained sections were evaluated with an Olympus IX81-DSU Spinning Disk confocal microscope using Slidebook software (Olympus, Center Valley, PA). In order to avoid bias, detection of Pg was performed by a single examiner (LR) blinded to placental pathology and other clinical information. The entire section was examined for the presence of Pg. A specimen was considered positive when the two serial sections showed a clear fluorescent signal for the Pg antigen.

A semi-quantitative scoring system was developed to quantify the Pg antigen load per patient. Pg specimens that were negative on all examined sections were assigned a score of 0. Specimens with one aggregate of Pg antigen per section were considered scant staining and were assigned a score of 1. Sections that had more than one Pg aggregate in at least one of the sections examined were considered moderate antigen load and assigned a score of 2. Sections with more than one Pg aggregate on all sections examined were considered heavy antigen load and assigned a score of 3.

### Statistical analysis

This study was executed in two stages. Preterm placenta and cord samples were analyzed in the first stage. Chi-square test was used to compare the number of Pg positive samples within the preterm cohort based on gestational age and adverse pregnancy characteristic. Dunnett’s post-hoc analysis was used to compare each pathology group to the preterm reference group. Logistic regression analyses of the preterm cohort were also performed to evaluate the independent effects of gestational age (in weeks), specific preterm pathology (HC, HCF, PE, PE+HELLP), SGA, exposure to antenatal steroids (full course) and mode of delivery (vaginal vs caesarean section) on the odds of having Pg in the preterm placenta and/or cord.

In addition, we assessed Pg density according to specific preterm pathology using the Kruskal-Wallis test. We conducted a cumulative odds ordinal logistic regression with proportional odds to determine whether specific pregnancy and delivery characteristics were independently associated with Pg density in the placenta and umbilical cord. The assumption of proportional odds was met for the ordinal regression models for Pg density in the placenta and the umbilical cord, and both models were a good fit to the observed data.

Using results from the preterm cohort, we performed a power-analysis (alpha of 0.05 and a power ≥ 0.80) to determine the sample size of term placentas needed. Based on this calculation, we included placental samples from 35 healthy term births in consecutive order of delivery (Fig 1).

Statistics were computed using SPSS 21.0 software (SPSS, Inc., Chicago, IL). Differences between groups were considered statistically significant at a two-sided probability value of <0.05.

## Results

### Pg prevalence in preterm placenta but not umbilical cord is linked to shorter gestation

**Fig 2:**
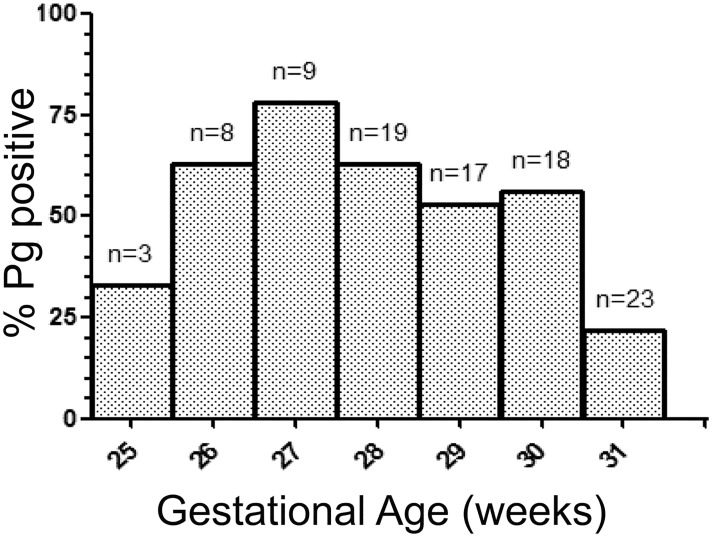
The distribution of Pg positive placental sections according to gestational age.

In the preterm cohort, Pg was present in 49 of 97 placental sections (51%). Of the positive samples, the distribution of scant, moderate, and heavy staining for Pg antigen was: 9 of 49 (18%), 30 of 49 (61%), and 10 of 49 (20%) respectively. Overall, the gestational length of preterm Pg positive placental specimens was shorter than Pg negative specimens, with mean gestational age ± SD being 28.8 ± 1.6 weeks vs. 29.8 ± 1.8 weeks respectively (P = 0.008). We next grouped the frequency of Pg positive placental sections according to gestational age (weeks) at delivery (Fig 2). The proportion of Pg positive placentas was highest at 27 weeks gestation (78%), after which the prevalence gradually decreased as gestational age increased: adjusted odds ratio (aOR) of 0.63 (95%CI: 0.48–0.85; p = 0.002) per week (Table 2). In line with these results, increasing gestational age (expressed in weeks) was less likely to have a heavy Pg density in the placenta, [aOR of 0.69 (95% CI: 0.53–0.89; p = 0.004)] (Table 3, S2a Fig). Pg positivity within the placenta was 4.02 times more likely to require delivery via caesarean section [aOR of 4.02 (95%CI: 1.15–14.04; p = 0.03)] (Table 2) and only 25% of caesarean sections were likely to have heavy Pg density in the placenta, [aOR of 0.25 (0.08–0.76; p = 0.01)] (Table 3). In contrast, there were no independent associations between antenatal steroid treatment or SGA and presence of Pg or Pg density in the placenta (Tables 2 and 3).

**Table 2:** Multivariable analyses of association between tissue Pg positivity and pregnancy and delivery characteristics in preterm cases.

	Pg placenta and/or cord		Pg cord		Pg placenta	
	aOR (95%CI)	*P* value	aOR (95%CI)	*P* value	aOR (95%CI)	*P* value
Gestational age (per week increase)	0.68 (0.51–0.91)	0.01	1.11 (0.85–1.45)	0.44	0.63 (0.48–0.85)	0.002
SGA	1.09 (0.28–4.23)	0.90	0.75 (0.22–2.51)	0.64	1.23 (0.36–4.20)	0.74
Full course of antenatal steroids	1.30 (0.44–3.80)	0.63	0.98 (0.35–2.74)	0.97	1.39 (0.48–4.00)	0.54
Caesarean section	0.74 (0.23–2.39)	0.62	0.46 (0.14–1.54)	0.21	4.02 (1.15–14.04)	0.03
Pathology group (ref = no pathology)						
HC	0.55 (0.12–2.58)	0.45	0.39 (0.08–1.92)	0.25	0.65 (0.13–3.22)	0.60
HCF	0.57 (0.12–2.61)	0.47	1.04 (0.24–4.49)	0.96	0.49 (0.10–2.28)	0.36
PE	3.63 (0.55–24.06)	0.18	6.73 (1.31–34.67)	0.02	1.31 (0.25–6.95)	0.76
PE+HELLP	1.81 (0.39–8.29)	0.45	1.85 (0.44–7.78)	0.40	0.55 (0.13–2.41)	0.43

Results from logistic regression models. Abbreviations: Pg, *Porphyromonas gingivalis*; aOR, adjusted odds ratio; CI, confidence interval; SGA, small for gestational age; HC, histologic chorioamnionitis; HCF, histologic chorioamnionitis with funisitis; PE, preeclampsia; HELLP, hemolysis, elevated liver enzymes and low platelet count.

**Table 3:** Multivariable analyses of association between tissue Pg density and pregnancy and delivery characteristics in preterm cases.

	Pg density placenta		Pg density umbilical cord	
	aOR (95%CI)	*P* value	aOR (95%CI)	*P* value
Gestational age (per week increase)	0.69 (0.53–0.89)	0.004	1.10 (0.85–1.42)	0.47
SGA	0.88 (0.31–2.50)	0.81	1.03 (0.35–3.05)	0.96
Full course of antenatal steroids	0.60 (0.23–1.53)	0.28	0.83 (0.32–2.19)	0.71
Caesarean section	0.25 (0.08–0.76)	0.01	2.07 (0.65–6.57	0.22
Pathology group (ref = no pathology)				
HC	1.60 (0.38–6.66)	0.52	3.06 (0.64–14.77)	0.16
HCF	1.75 (0.45–6.89)	0.42	1.10 (0.28–4.34)	0.89
PE	1.27 (0.32–5.07)	0.74	0.26 (0.06–1.11)	0.07
PE+HELLP	2.13 (0.58–7.83)	0.26	0.64 (0.16–2.45)	0.51

Results from cumulative odds ordinal logistic regression with proportional odds models. Abbreviations: Pg, *Porphyromonas gingivalis*; aOR, adjusted odds ratio; CI, confidence interval; SGA, small for gestational age; HC, histologic chorioamnionitis; HCF, histologic chorioamnionitis with funisitis; PE, preeclampsia; HELLP, hemolysis, elevated liver enzymes and low platelet count.

Forty of 97 (41%) preterm umbilical cord sections were positive for Pg. Within the positive group, the distribution of scant, moderate and heavy staining for Pg antigen was: 6 of 40 (15%), 25 of 40 (62%), and 9 of 40 (23%) respectively. There was no independent association between the presence of Pg or Pg density within the umbilical cord and gestational age, antenatal steroid treatment, or mode of delivery (Tables 2 and 3).

Since we observed a significant association between Pg positivity in the placenta and shorter gestation length, we postulated that the proportion of Pg positive specimens in term pregnancy would be lower than in pregnancies ending prematurely. Because term specimens were not available for study in the Rotterdam cohort, we obtained archived term pregnancy specimens from a prospective cohort collected at Wilhelmina Children’s Hospital/University Medical Center Utrecht [24]. In the term cohort, Pg was detected in only 2 of the 35 (6%) placental sections. In both specimens the Pg antigen density was scant (score of 1). None of the term umbilical cord sections were positive for Pg.

### PE is associated with Pg positive status in the umbilical cord but not placenta

We next categorized preterm placental and umbilical cord data (n = 97) according to specific preterm pathology: HC (n = 18), HCF (n = 23), preeclampsia (n = 14), preeclampsia with HELLP-syndrome (n = 25) and a preterm reference group in which these diagnoses were all absent (n = 17). In brief, the reference group consisted of cases of spontaneous vaginal delivery without HC or HCF (n = 6) and cases of delivery via cesarean section based on medical indication (n = 11; fetal distress and/or intra-uterine growth restriction). S2 Table provides further description of the clinical characteristics of the five preterm subgroups. Pg positive status within the placenta was not different among the groups. However, multivariate analysis showed that PE was 6.73 times more likely to have Pg in the umbilical cord [OR 6.73 (95%CI: 1.31–36.67; p = 0.02)] but not the placenta (Table 3). There were no significant differences in Pg density in the placenta or in umbilical cords among the preterm subgroups (Tables 2 and 3). The distribution of Pg density in placental and cord sections according to preterm subgroup is summarized in S2b Fig and S3 Table.

### The *in situ* localization of Pg in placenta and umbilical cord specimens

**Fig 3:**
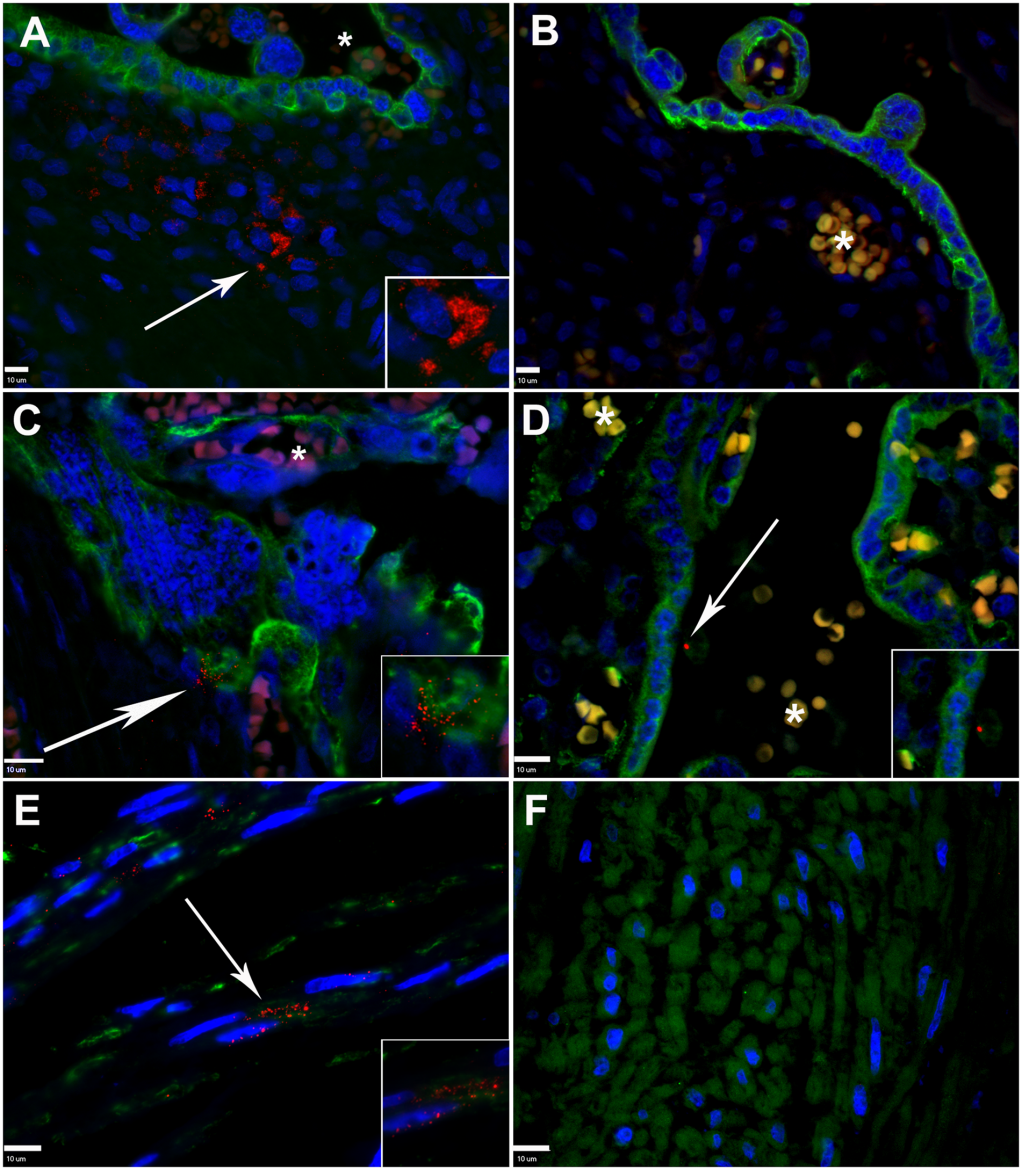
*In situ* detection of Pg in placental and umbilical cord sections from preterm and term tissues. **A.** Placental villous section from a preterm specimen demonstrating the distribution of Pg (red, white arrow) within the extracellular matrix. Cytotrophoblasts/syncytiotrophoblasts (green) were detected with cytokeratin-7 specific antibody. **B.** Corresponding preterm placental section stained with pre-adsorbed Pg-specific antiserum (red) and cytokeratin-7 specific antibody (green). **C.** Pre-term placental specimen demonstrating Pg (red, white arrow) attached to cytotrophoblasts/syncytiotrophoblasts (green). **D.** Term placental villus section demonstrating Pg (red, white arrow) in association with syncytiotrophoblast (green). **E.** Preterm umbilical cord section demonstrating extracellular and internalized Pg (white arrow) within the perivascular stroma, myofibroblast (green) were labelled with vimentin specific antibody. **F.** Representative umbilical cord section from a term specimen, which were negative for Pg. Cell nuclei (blue) were stained with DAPI. *Indicates autofluorescent red blood cells in maternal or fetal circulation. In all panels, magnified inserts are Pg positive regions demarcated by arrows. All scale bars (bottom left corner) are equivalent to 10 μm, images are 400x or 600x magnification.

Preterm placental sections that were incubated with Pg specific antiserum displayed distinct aggregates within the villous stroma that were not observed in sections incubated with pre-immune serum (Fig 3A and S1C Fig). These aggregates were most commonly observed within the extracellular matrix within the villous mesenchyme (Fig 3A and S1C Fig). On a few specimens, Pg was also detected in association with fetal capillaries (endothelial cells), data not shown. In order to confirm that these aggregates were specific for Pg, additional sections were incubated with Pg specific anti-serum that was pre-adsorbed with formalin-fixed Pg. Specimens incubated with pre-absorbed serum did not show the staining pattern that was detected in the corresponding placental specimens incubated with Pg specific antisera (Fig 3B). This confirmed that these aggregates contain Pg antigen. In 9 of 49 positive preterm placenta specimens (18%), Pg was also observed attached to cytotrophoblasts and/or syncytiotrophoblasts (Fig 3C). This pattern of colonization was observed in HC (n = 1), preeclampsia (n = 1), preeclampsia with HELLP-syndrome (n = 5) and preterm reference group (n = 2) cases, which ranged from 28 to 32 weeks gestation.

In term specimens positive for Pg, scant amounts of Pg antigen were detected in association with syncytiotrophoblasts (Fig 3D), but Pg was never detected within the villous mesenchyme.

In preterm umbilical cord sections Pg was primarily detected in the umbilical arterial intimal layer either in the extracellular matrix or appearing to be internalized in vimentin positive cells that resembled smooth muscle cells or fibroblast cells (Fig 3E). Pg was occasionally detected in Wharton’s jelly (S1C Fig) but not in the umbilical vessel lumen in any of cord specimens. This staining pattern was also confirmed to be Pg antigen (Fig 3F). Pg antigen was never detected in term umbilical cord specimens.

## Discussion

Our overall objective was to ascertain if the location of Pg within placental and/or umbilical cord sections from extremely preterm and very preterm infants (25 to 32 weeks gestation) was different from normal term deliveries. In addition, we wanted to determine if the prevalence or tissue distribution of Pg was associated with a particular obstetric complication such as HC, HCF, PE or PE with HELLP-syndrome. Similar to previous reports [15, 16, 18], we found a greater proportion of preterm placental specimens positive for Pg than normal term tissues. Also consistent with previous studies [16] was the detection of more Pg antigen in preterm specimens compared to term tissues. A novel finding in our study was that we detected a distinctive distribution of Pg antigen within the placenta and umbilical cord from preterm specimens that was not found in term tissues. Further, the unique distribution of Pg antigen in the villous stroma of preterm placental specimens was linked to shorter gestation length and the need for cesarean section. Another novel finding was the association between PE and the presence of Pg antigens in the umbilical cord. Our results suggest that the location of the microbe within the placenta or umbilical cord may be an important feature in Pg-associated adverse pregnancy outcome.

We can only speculate as to how Pg gains entry into the placental mesenchyme. One possible explanation could be that Pg managed to cross from the maternal circulation into the fetal tissues through the syncytiotrophoblast. This is the multi-nucleated layer of fused cytotrophoblasts cells covering the entire villous tree, thereby separating the maternal and fetal compartments of the placenta [29]. However, the syncytiotrophoblast possesses robust defense mechanisms against microbial invasion (e.g. the lack of intercellular junctions and receptors, and resistance to receptor-independent invasion) [30–32]. Vertical transmission of microbes through this route is therefore unlikely [31]. An exception would be a disruption in the syncytiotrophoblast layer forming a direct *port d’entrée*. Alternatively, Pg may invade the placenta through the decidua, at the uterine-trophoblast interface [31]. Here, microbes may spread from the maternal circulation to susceptible extra-villous trophoblast cells by direct invasion or cell-to-cell spread during implantation of the trophoblast into the uterine wall in the first trimester of pregnancy [31, 32]. Pg efficiently invades a variety of host cell types [13, 33] including extravillous trophoblast cells [34]. Moreover, internalized Pg can escape the host cell and invade surrounding cells [35], demonstrating that dissemination of Pg through the uterine-trophoblast interface into the placental mesenchyme is a plausible route of infection.

The association of Pg in the placenta with shorter gestation and cesarean section supports the hypothesis that the microbe in this location has a detrimental effect on placental function or pregnancy. Surprisingly, this pattern of colonization was found in a variety of obstetric complications that involve different utero-placental pathologies and clinical manifestations (PE vs. HC and HCF). One possible explanation for this finding is the genetic heterogeneity among Pg strains and its impact on microbial virulence and disease outcome [14, 36]. Both *in vitro* and *in vivo* studies have demonstrated that distinct Pg strains display different behaviors in the host and varying pathogenic potential [21, 26, 37, 38]. During pregnancy, rodents infected with Pg strain A7436 develop uterine vasculitis, utero-placental thrombosis/hemorrhage, structural alterations within the placental vascular layer (labyrinth), and fetal growth restriction [19–21]; pathology that is more consistent with PE [39, 40]. On the other hand, C57BL/6 mice infected with Pg strain W83 exhibit pathology that is more consistent with HC and HCF such as disruption of the integrity of the chorioamnion, chorioamnionitis, and spontaneous preterm birth [22].

An intriguing possibility is that Pg may be acting as a keystone species of HC and HCF by modulating host antimicrobial defenses at the maternal fetal interface in a manner that facilitates invasion and overgrowth of other microbes within the fetal compartment. There is presumptive evidence that Pg can act as a keystone species at extra-oral tissue sites, including the maternal-fetal interface. In a subcutaneous abscess model of polymicrobial infection, Pg suppresses neutrophil-mediated microbial killing, which enhances the survival of *Fusobacterium nucleatum* without reducing inflammation [41]. In co-infection studies with *Campylobacter rectus*, the presence of Pg modulates the placental response to *C*. *rectus* by reducing placental expression of toll-like receptor 4 expression [42], which did not reduce adverse pregnancy outcome. Rather, co-infected animals displayed decreased fecundity and fetal growth restriction that did not occur in animals infected with *C*. *rectus* alone [42].

PE was the only obstetric complication to show a significant association with Pg in the umbilical cord. This association may be an extension of what we detected in the placenta since most PE cases that were positive in the cord were also positive in the placenta. In this preterm cohort Pg was most commonly detected within the intimal layer of the umbilical artery. Since this vessel is returning blood from the fetus to the placenta, it is unclear at this time what impact the presence of Pg in the umbilical cord may have on the fetus.

Our study has several limitations. Although our focus was specifically on Pg, we recognize that placental infections are often polymicrobial [15, 17, 18], and polymicrobial interactions within the utero-fetal compartment may result in an different outcome than a monotypic infection [42]. Pg has been detected coexisting with other bacteria in the placenta [15, 17, 18], amniotic fluid [43], and neonatal gastric aspirates [44] from complicated pregnancies. Therefore, we cannot discount the possibility that HC and HCF in particular, may be a consequence of other bacterial species coexisting with Pg in the placental stroma or present in the amniotic cavity [45, 46]. Despite this limitation we observed a strong association between the presence of Pg within placental villous mesenchyme and shorter gestation as well as cesarean section. This association, along with supportive findings in rodent infection studies [19–21, 47] suggest the microbe may be exerting a detrimental effect on placental function regardless of what other microbes may be present in the environment.

Another limitation was that tissues from term pregnancies at the Erasmus Medical Center-Sophia Children’s Hospital, in Rotterdam were not available for comparison with the preterm tissues. This is why term specimens from Wilhelmina Children’s Hospital in Utrecht were included in this study. Although we confirmed that maternal age, gravidity, and parity were the same in both cohorts, we cannot rule out the possibility that differences we observed between preterm and term groups could be due to inherent differences in these populations. In addition, estimates of gestational age were extracted from the obstetric record at the time of birth, and information of the method of ascertainment was lacking at the individual level. In cases where gestational age was estimated by using the last menstrual period, a small degree of misclassification may have influenced our findings, although the impact is likely to have been small [48, 49].

In summary, our study provides a novel insight into the relevance of Pg in obstetric complications that lead to preterm delivery. Specifically, we found that the presence of Pg within the placental villous stroma was a unique feature of very preterm delivery irrespective of underlying pathology associated with prematurity. We also found an association between the presence of Pg in the umbilical cord and PE. Given that monotypic infection of the placenta with Pg promotes intrauterine pathology and fetal growth restriction in experimental models [19, 21, 42], our findings suggest that invasion of the villous or umbilical cord stroma by Pg alone or in combination with other bacteria, may be a critical factor in the pathogenesis of Pg-associated adverse pregnancy outcome.

## Supporting Information

S1 Fig Validation of the specificity of anti-Pg W83 and efficacy for use with formalin fixed paraffin embedded tissues. **A.** The specificity of the antiserum to Pg was confirmed by immunoblotting against a panel of Gram negative bacteria and human cell lines: Lane 1, Pg strain W83 (positive control); Lane 2, *Fusobacterium nucleatum*; Lane 3, *Prevotella intermedia;* Lane 4, *Escherichia coli;* Lane 5, *Proteus mirabilis;* Lane 6, *Klebsiella pneumonia;* Lane 7, HTR8 trophoblast cells; Lane 8, BPH-1 cells; Lane 9, human umbilical vein endothelial cells; Lane 10, Hela cells; Lane 11, fibroblasts; Lane 12 blank. Immunoblotting was performed with a 96 well membrane-bottom filter plate- vacuum apparatus. Briefly, a PVDF membrane was loaded into the apparatus, and 400 μl of phosphate buffered saline with 0.05% Tween-20 (PBS-Tween) was passed through to soak the membrane. Each well was loaded with 200 μls of bacterial suspension (10^7^ CFU/ml) or cell lysate (10^5^ cells/ ml), in duplicate. The fluid was pulled through the PVDF membrane by vacuum. The membrane was then blocked with Starting Block^™^ (TBS) blocking buffer (Thermo Scientific, Rockford, IL) for 30 minutes at room temperature. Pg specific rabbit serum and the corresponding pre-immune serum were diluted 1:2000 in TBS blocking buffer. Each well received 200 μl aliquots of the diluted Pg antiserum (top row) or pre-immune serum (bottom row), and incubated at room temperature for 2 hours. The membrane was removed from the apparatus and washed 4 times with PBS-Tween. The entire membrane was then immersed in goat anti-rabbit–alkaline-phosphate labeled antibody (NOVEX^®^, Life Technologies, Frederick, MD) that was diluted 1:2000 in TBS Blocking buffer. After 30 minutes at room temperature, the membrane was washed 4 times with PBS-Tween. The washed membrane was then immersed in NOVEX^®^ AP-chromogenic substrate (Life Technologies, Frederick, MD), and incubated at room temperature until a strong, distinctive purple signal was observed in the positive control well (Lane 1). The membrane was washed once with PBS-Tween and allowed to air dry. **B.** Representative images of a preterm placental section stained with anti-Pg W83 (red, white arrow) and anti-cytokeratin 7 (green) or with corresponding isotype controls. Nuclei (blue) were stained with DAPI. Images are 600x magnification. Immunofluorescent stainings were performed as already described in the methods section of the manuscript.
(PDF)

S2 Fig The distribution of Pg density in the Pg positive placental and umbilical cord sections according to gestational age in weeks (A) and specific preterm pathology (B). *Porphyromonas gingivalis* (Pg) density was scored using a semi-quantitive scale: negative, scant, moderate, and heavy. Reference group (n = 17) refers to preterm specimens that did not have a histological or clinical diagnosis of HC (n = 18), HCF (n = 23), PE (n = 14), or PE + HELLP (n = 25). Abbreviations: HC, histologic chorioamnionitis; HCF, histologic chorioamnionitis with funisitis; PE, preeclampsia; PE+HELLP, preeclampsia with hemolysis, elevated liver enzymes, and low platelet count.
(PDF)

S1 Table Clinical characteristics of in- and excluded preterm subjects. Numbers represent number and percentage of newborns or mothers in which characteristic is present for categorical data; median and interquartile range for ordinal data; and mean ± SD for continuous data. Differences between groups were tested using Chi-square, Mann-Whitney U and Student t-test, respectively. Abbreviations used: PPROM, preterm premature rupture of membranes; HC, histological chorioamnionitis; HCF, histological chorioamnionitis with funisitis; HELLP, hemolysis, elevated liver enzymes and low platelet count; SGA, small for gestational age
(PDF)

S2 Table Clinical characteristics preterm subjects according to underlying pathology. Numbers represent number and percentage of newborns or mothers in which characteristic is present for categorical data; median and interquartile range for ordinal data; and mean ± SD for continuous data. Differences between groups were tested using Chi-square, Kruskal Wallis and ANOVA, respectively. Dunnett’s post-hoc analysis was used to compare each group to the reference group. Significant differences are indicated by: *P < 0.05; **P < 0.01; and ***P < 0.001. Abbreviations used: HC, histological chorioamnionitis; HCF, with funisitis; PE, preeclampsia; HELLP, hemolysis, elevated liver enzymes and low platelet count; PPROM, preterm premature rupture of membranes; SGA, small for gestational age. a) The reference group consists of all preterm deliveries in which there was no HC, HCF, PE or PE with HELLP-syndrome.
(PDF)

S3 Table Median Pg density values in preterm placentas and cords. *Porphyromonas gingivalis* (Pg) density was scored using a semi-quantitative scale: negative, scant, moderate, and heavy. Numbers represent the median Pg density score per group with interquartile range. Differences in Pg density between groups were tested using Kruskal-Wallis test. Abbreviations: HC, histologic chorioamnionitis; HCF, histologic chorioamnionitis with funisitis; PE, preeclampsia; HELLP, hemolysis, elevated liver enzymes and low platelet count.
(PDF)
