# Supplementary material for: Porphyromonas gingivalis within Placental Villous Mesenchyme and Umbilical Cord Stroma Is Associated with Adverse Pregnancy Outcome
Source: PLoS One. 2016 Jan 5;11(1):e0146157. doi: 10.1371/journal.pone.0146157 (PMC4701427; doi:10.1371/journal.pone.0146157)
Supplement: S1 Fig — A. The specificity of the antiserum to Pg was confirmed by immunoblotting against a panel of Gram negative bacteria and human cell lines: Lane 1, Pg strain W83 (positive control); Lane 2, Fusobacterium nucleatum; Lane 3, Prevotella intermedia; Lane 4, Escherichia coli; Lane 5, Proteus mirabilis; Lane 6, Klebsiella pneumonia; Lane 7, HTR8 trophoblast cells; Lane 8, BPH-1 cells; Lane 9, human umbilical vein endothelial cells; Lane 10, Hela cells; Lane 11, fibroblasts; Lane 12 blank. Immunoblotting was performed with a 96 well membrane-bottom filter plate- vacuum apparatus. Briefly, a PVDF membrane was loaded into the apparatus, and 400 μl of phosphate buffered saline with 0.05% Tween-20 (PBS-Tween) was passed through to soak the membrane. Each well was loaded with 200 μls of bacterial suspension (107 CFU/ml) or cell lysate (105 cells/ ml), in duplicate. The fluid was pulled through the PVDF membrane by vacuum. The membrane was then blocked with Starting Block™ (TBS) blocking buffer (Thermo Scientific, Rockford, IL) for 30 minutes at room temperature. Pg specific rabbit serum and the corresponding pre-immune serum were diluted 1:2000 in TBS blocking buffer. Each well received 200 μl aliquots of the diluted Pg antiserum (top row) or pre-immune serum (bottom row), and incubated at room temperature for 2 hours. The membrane was removed from the apparatus and washed 4 times with PBS-Tween. The entire membrane was then immersed in goat anti-rabbit–alkaline-phosphate labeled antibody (NOVEX®, Life Technologies, Frederick, MD) that was diluted 1:2000 in TBS Blocking buffer. After 30 minutes at room temperature, the membrane was washed 4 times with PBS-Tween. The washed membrane was then immersed in NOVEX® AP-chromogenic substrate (Life Technologies, Frederick, MD), and incubated at room temperature until a strong, distinctive purple signal was observed in the positive control well (Lane 1). The membrane was washed once with PBS-Tween and allowed to air dry. B. Representative [file pone.0146157.s001.pdf]

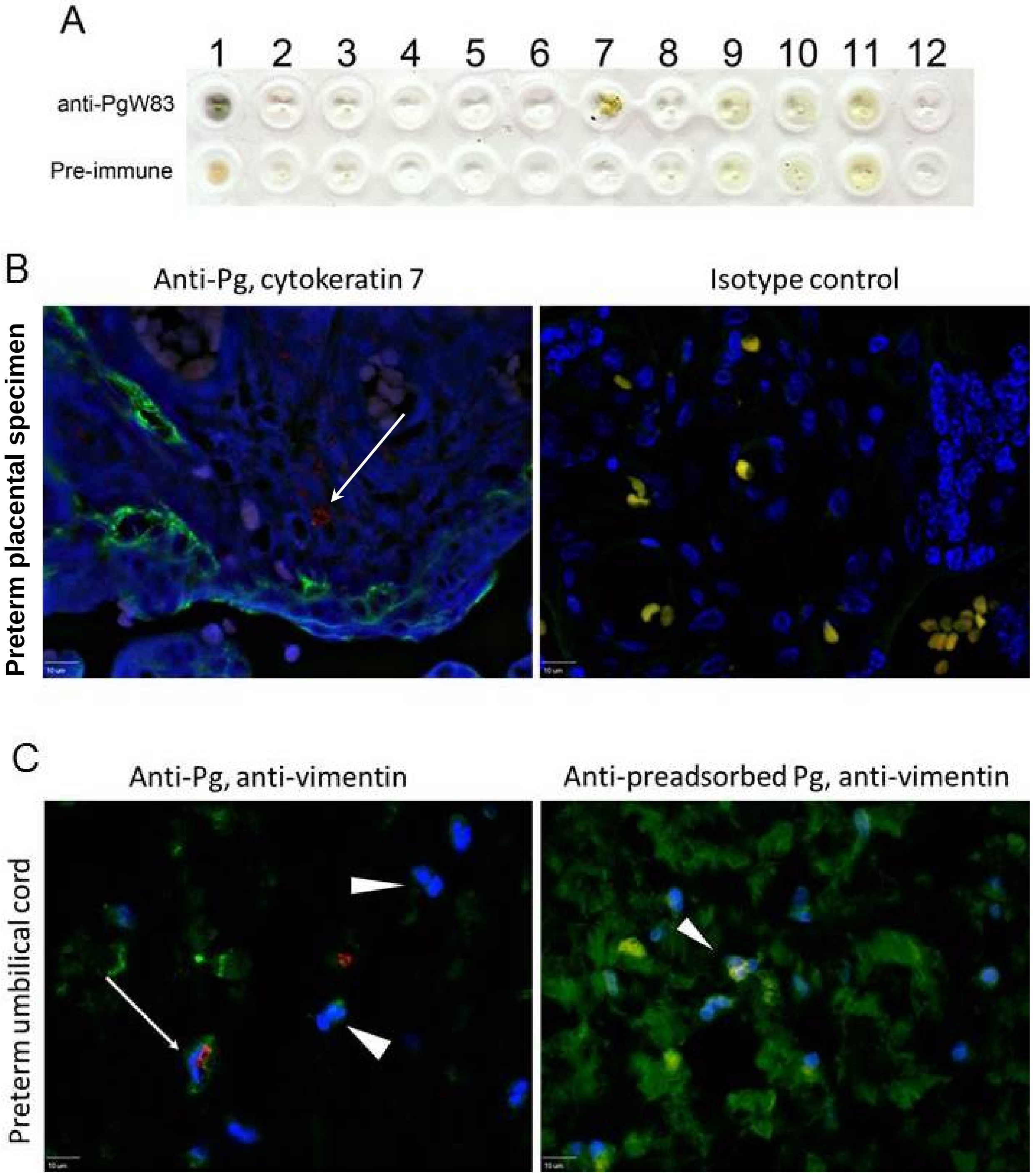

Fig. S1. Validation of the specificity of anti-Pg W83 and efficacy for use with formalin fixed paraffin embedded tissues.

A. The specificity of the antiserum to Pg was confirmed by immunoblotting against a panel of Gram negative bacteria and human cell lines: Lane 1, Pg strain W83 (positive control);

Lane 2, *Fusobacterium nucleatum*; Lane 3, *Prevotella intermedia;* Lane 4, *Escherichia coli;* Lane 5, *Proteus mirabilis;* Lane 6, *Klebsiella pneumonia;* Lane 7, HTR8 trophoblast cells; Lane 8, BPH-1 cells; Lane 9, human umbilical vein endothelial cells; Lane 10, Hela cells; Lane 11, fibroblasts; Lane 12 blank.

Immunoblotting was performed with a 96 well membrane-bottom filter plate- vacuum apparatus. Briefly, a PVDF membrane was loaded into the apparatus, and 400 µl of phosphate buffered saline with 0.05% Tween-20 (PBS-Tween) was passed through to soak the membrane. Each well was loaded with 200 µls of bacterial suspension (10^7 CFU/ml) or cell lysate (10^5 cells/ml), in duplicate. The fluid was pulled through the PVDF membrane by vacuum. The membrane was then blocked with Starting Block™ (TBS) blocking buffer (Thermo Scientific, Rockford, IL) for 30 minutes at room temperature. Pg specific rabbit serum and the corresponding pre-immune serum were diluted 1:2000 in TBS blocking buffer. Each well received 200 µl aliquots of the diluted Pg antiserum (top row) or pre-immune serum (bottom row), and incubated at room temperature for 2 hours. The membrane was removed from the apparatus and washed 4 times with PBS-Tween. The entire membrane was then immersed in goat anti-rabbit –alkaline-phosphate labeled antibody (NOVEX®, Life Technologies, Frederick, MD) that was diluted 1:2000 in TBS Blocking buffer. After 30 minutes at room temperature, the membrane was washed 4 times with PBS-Tween. The washed membrane was then immersed in NOVEX® AP-chromogenic substrate (Life Technologies, Frederick, MD), and incubated at room temperature until a strong, distinctive purple signal was observed in the positive control well (Lane 1). The membrane was washed once with PBS-Tween and allowed to air dry.

B. Representative images of a preterm placental section stained with anti-Pg W83 (red, white arrow) and anti-cytokeratin 7 (green) or with corresponding isotype controls. Nuclei (blue) were stained with DAPI. Images are 600x magnification. Immunofluorescent stainings were performed as already described in the methods section of the manuscript.

C. Representative umbilical cord section from a preterm case with evidence of funisitis (neutrophils demarcated with arrowheads). One section was incubated with anti-Pg antibody (red, long arrow) and anti-vimentin (green) antibody. The corresponding section (right panel) was incubated with Pg antiserum that was first pre-adsorbed with formalin fixed Pg and anti-vimentin antibody (green). Nuclei (blue) were stained with DAPI. Images are 600x magnification.

Adsorption of Pg specific antibodies from the rabbit serum was performed by incubating the serum with formalin fixed whole Pg strain W83. Pg was first fixed by incubating whole bacteria in 4% buffered formalin and incubated for 15 minutes at room temperature before washing with sterile phosphate buffered saline. Fixed bacteria were washed three times in order to remove any residual fixative before adding to undiluted Pg specific rabbit serum. Adsorption was performed by adding 10 µl of undiluted rabbit antiserum to a pellet of Pg that contained approximately 10^9 CFU of Pg and incubated overnight at 40ºC. The suspension was then centrifuged at 16,000 x g to remove bacteria. The supernatant was carefully collected and the bacterial pellet was discarded. In order to ensure that the majority of Pg specific antibodies were removed, the procedure was repeated two additional times using the same number of formalin-fixed bacteria.
