# Supplementary material for: Porphyromonas gingivalis within Placental Villous Mesenchyme and Umbilical Cord Stroma Is Associated with Adverse Pregnancy Outcome
Source: PLoS One. 2016 Jan 5;11(1):e0146157. doi: 10.1371/journal.pone.0146157 (PMC4701427; doi:10.1371/journal.pone.0146157)
Supplement: S2 Fig — Porphyromonas gingivalis (Pg) density was scored using a semi-quantitive scale: negative, scant, moderate, and heavy. Reference group (n = 17) refers to preterm specimens that did not have a histological or clinical diagnosis of HC (n = 18), HCF (n = 23), PE (n = 14), or PE + HELLP (n = 25). Abbreviations: HC, histologic chorioamnionitis; HCF, histologic chorioamnionitis with funisitis; PE, preeclampsia; PE+HELLP, preeclampsia with hemolysis, elevated liver enzymes, and low platelet count. (PDF) [file pone.0146157.s002.pdf]

A.

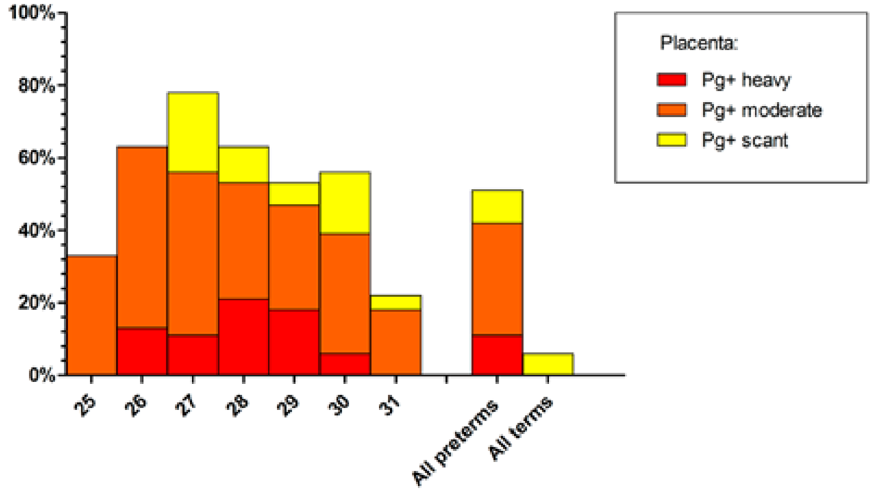

B.

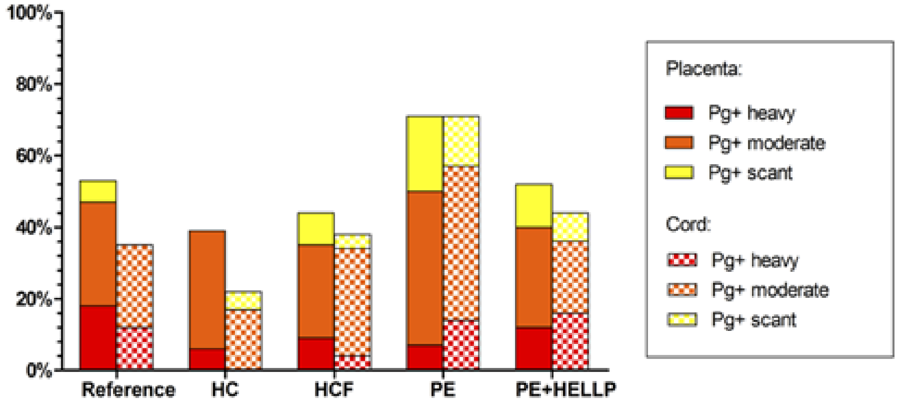

Fig. S2. The distribution of Pg density in the Pg positive placental and umbilical cord sections according to gestational age in weeks (A) and specific preterm pathology (B).

Porphyromonas gingivalis (Pg) density was scored using a semi-quantitive scale: negative, scant, moderate, and heavy. Reference group (n=17) refers to preterm specimens that did not have a histological or clinical diagnosis of HC (n=18), HCF (n=23), PE (n=14), or PE + HELLP (n=25). Abbreviations: HC, histologic chorioamnionitis; HCF, histologic chorioamnionitis with funisitis; PE, preeclampsia; PE+HELLP, preeclampsia with hemolysis, elevated liver enzymes, and low platelet count.
