# Supplementary material for: Porphyromonas gingivalis within Placental Villous Mesenchyme and Umbilical Cord Stroma Is Associated with Adverse Pregnancy Outcome
Source: PLoS One. 2016 Jan 5;11(1):e0146157. doi: 10.1371/journal.pone.0146157 (PMC4701427; doi:10.1371/journal.pone.0146157)
Supplement: S1 Table — Numbers represent number and percentage of newborns or mothers in which characteristic is present for categorical data; median and interquartile range for ordinal data; and mean ± SD for continuous data. Differences between groups were tested using Chi-square, Mann-Whitney U and Student t-test, respectively. Abbreviations used: PPROM, preterm premature rupture of membranes; HC, histological chorioamnionitis; HCF, histological chorioamnionitis with funisitis; HELLP, hemolysis, elevated liver enzymes and low platelet count; SGA, small for gestational age (PDF) [file pone.0146157.s003.pdf]

Table S1. Clinical characteristics of in- and excluded preterm subjects

		Included preterm singletons (n=97)	Excluded preterm singletons (n=122)	*P* value
**Maternal characteristics**	Maternal age (years)	31±6	30±6	0.51
	Gravidity	2 (1-3)	1 (1-2)	0.02
	Parity	0 (0-1)	0 (0-1)	0.01
**Pregnancy & delivery characteristics**	Gestational age (weeks)	29±2	29±2	0.90
	Full course antenatal steroids	70 (72%)	88 (72%)	1.00
	PPROM	26 (27%)	34 (28%)	0.86
	Caesarean section	65 (67%)	78 (64%)	0.64
	Placenta weight (grams)	260±80	258±118	0.90
	Preterm reference group	17 (18%)	28 (23%)	
	HC	18 (19%)	14 (11%)	
	HCF	23 (24%)	28 (23%)	
	Preeclampsia	14 (14%)	20 (16%)	
	Preeclampsia+HELLP	25 (26%)	32 (26%)	0.60
**Infant characteristics**	Male gender	56 (58%)	58 (48%)	0.13
	Birth weight (grams)	1145±343	1135±371	0.42

SGA	23 (24%)	43 (35%)	0.07
In-hospital mortality	13 (13%)	10 (8%)	0.21

Numbers represent number and percentage of newborns or mothers in which characteristic is present for categorical data; median and interquartile range for ordinal data; and mean ± SD for continuous data. Differences between groups were tested using Chi-square, Mann-Whitney U and Student t-test, respectively. Abbreviations used: PPROM, preterm premature rupture of membranes; HC, histological chorioamnionitis; HCF, histological chorioamnionitis with funisitis; HELLP, hemolysis, elevated liver enzymes and low platelet count; SGA, small for gestational age.
