# Supplementary material for: Porphyromonas gingivalis within Placental Villous Mesenchyme and Umbilical Cord Stroma Is Associated with Adverse Pregnancy Outcome
Source: PLoS One. 2016 Jan 5;11(1):e0146157. doi: 10.1371/journal.pone.0146157 (PMC4701427; doi:10.1371/journal.pone.0146157)
Supplement: S2 Table — Numbers represent number and percentage of newborns or mothers in which characteristic is present for categorical data; median and interquartile range for ordinal data; and mean ± SD for continuous data. Differences between groups were tested using Chi-square, Kruskal Wallis and ANOVA, respectively. Dunnett’s post-hoc analysis was used to compare each group to the reference group. Significant differences are indicated by: *P < 0.05; **P < 0.01; and ***P < 0.001. Abbreviations used: HC, histological chorioamnionitis; HCF, with funisitis; PE, preeclampsia; HELLP, hemolysis, elevated liver enzymes and low platelet count; PPROM, preterm premature rupture of membranes; SGA, small for gestational age. a) The reference group consists of all preterm deliveries in which there was no HC, HCF, PE or PE with HELLP-syndrome. (PDF) [file pone.0146157.s004.pdf]

Table S2. Clinical characteristics preterm subjects according to underlying pathology

	Reference[a] (n=17)	HC (n=18)	HCF (n=23)	PE (n=14)	PE+HELLP (n=25)	*P* value
**Maternal characteristics**						
Maternal age (years)	33±5	28±5	31±6*	31±6	30±5	0.14
Gravidity	2 (1-3)	2 (1-3)	2 (1-5)	2 (1-4)	1 (1-3)	0.34
Parity	0 (0-1)	0 (0-1)	1 (0-2)	1 (0-2)	0 (0-1)	0.42
**Pregnancy & delivery characteristics**						
Gestational age (weeks)	30±2	29±2	28±2*	30±1	30±2	0.028
Full course antenatal steroids	10 (59%)	13 (72%)	15 (65%)	9 (64%)	23 (92%)	0.12
PPROM	2 (12%)	7 (39%)	17 (74%)***	0	0	< 0.001
Caesarean section	11 (65%)	7 (39%)	9 (39%)	14 (100%)	24 (96%)*	< 0.001
Placenta weight (grams)	242±92	315±86*	294±64	201±71	233±74	< 0.001
**Infant characteristics**						
Male gender	8 (47%)	14 (78%)	14 (61%)	8 (57%)	12 (48%)	0.31
Birth weight (grams)	1138±322	1396±367	1188±309	999±355	1078±278	0.007
SGA	7 (41%)	0**	1 (4%)*	8 (57%)	7 (38%)	< 0.001
In-hospital mortality	2 (12%)	2 (11%)	6 (26%)	0	3 (12%)	0.24

Numbers represent number and percentage of newborns or mothers in which characteristic is present for categorical data; median and interquartile range for ordinal data; and mean ± SD for continuous data. Differences between groups were tested using Chi-square, Kruskal Wallis and ANOVA, respectively. Dunnett's post-hoc analysis was used to compare each group to the reference group. Significant differences are indicated by: $^{*}P < 0.05$; $^{**}P < 0.01$; and $^{***}P < 0.001$. Abbreviations used: HC, histological chorioamnionitis; HCF, with funisitis; PE, preeclampsia; HELLP, hemolysis, elevated liver enzymes and low platelet count; PPROM, preterm premature rupture of membranes; SGA, small for gestational age.

a) The reference group consists of all preterm deliveries in which there was no HC, HCF, PE or PE with HELLP-syndrome.
