# Supplementary material for: Porphyromonas gingivalis within Placental Villous Mesenchyme and Umbilical Cord Stroma Is Associated with Adverse Pregnancy Outcome
Source: PLoS One. 2016 Jan 5;11(1):e0146157. doi: 10.1371/journal.pone.0146157 (PMC4701427; doi:10.1371/journal.pone.0146157)
Supplement: S3 Table — Porphyromonas gingivalis (Pg) density was scored using a semi-quantitative scale: negative, scant, moderate, and heavy. Numbers represent the median Pg density score per group with interquartile range. Differences in Pg density between groups were tested using Kruskal-Wallis test. Abbreviations: HC, histologic chorioamnionitis; HCF, histologic chorioamnionitis with funisitis; PE, preeclampsia; HELLP, hemolysis, elevated liver enzymes and low platelet count. (PDF) [file pone.0146157.s005.pdf]

Table S3. Median Pg density values in preterm placentas and cords

		Preterm pathology subgroups					
		Reference group (n=17)	HC (n=18)	HCF (n=22)	PE (n=14)	PE+HELLP (n=25)	P-value
**Pg density**	Placenta	1 (0-2)	0 (0-2)	0 (0-2)	2 (0-2)	1 (0-2)	0.67
	Umbilical cord	0 (0-2)	0 (0-0)	0 (0-2)	2 (0-2)	0 (0-2)	0.10

Porphyromonas gingivalis (Pg) density was scored using a semi-quantitative scale: negative, scant, moderate, and heavy. Numbers represent the median Pg density score per group with interquartile range. Differences in Pg density between groups were tested using Kruskal-Wallis test. Abbreviations: HC, histologic chorioamnionitis; HCF, histologic chorioamnionitis with funisitis; PE, preeclampsia; HELLP, hemolysis, elevated liver enzymes and low platelet count.
